# Supplementary material for: Tackling Missing Heritability by Use of an Optimum Curve: A Systematic Review and Meta-Analysis
Source: Int J Mol Sci. 2019 Oct 15;20(20):5104. doi: 10.3390/ijms20205104 (PMC6829377; doi:10.3390/ijms20205104)
Supplement: Supplementary file 1 [file ijms-20-05104-s001.pdf]

Tackling Missing Heritability by Use of an Optimum Curve: a Systematic Review and Meta-Analysis

SUPPLEMENTARY MATERIAL

Search Results – Details

- Out of the 36 full-text articles that were assessed for eligibility, 22 articles reported family-based studies on the genetic association between the short/long 5-HTTLPR polymorphism and autism spectrum disorder (ASD). One of these articles [1] reported two separate family-based studies on samples from two different continents. Hence, we had 23 family based genetic association studies at our disposal. Five of these studies did not meet our selection criteria: the study by Valencia et al. (2012) [2] because it was written in Spanish, the study by Coutinho et al. (2007) [3] because upon request, the authors informed us that the sample of this study was a subset of the sample of a previous study by Coutinho et al. (2004) [4] that was also selected, and the studies by Ramoz et al. (2006) [5], Longo et al. (2009) [6] and Jaiswal et al. (2015) [7] because the reported data did not allow for calculation of the odds ratio (OR) of transmission of heterozygous parents to individuals with ASD of the short relative to the long 5-HTTLPR variant. Therefore, 17 articles, reporting on 18 family based genetic association studies, were included in our meta-analysis. The remaining 19 articles – among which 5 family based and 8 population based association studies – were excluded.
- The study by McCauley et al. (2004) [8] did not report the numbers of short and long 5-HTTLPR variants, transmitted and not transmitted – respectively – from heterogeneous parents to affected offspring. For this study, we used the data that Huang & Santangelo (2008) [9] reported after receiving them from the authors.
- In the study by Mulder et al. (2005) [10], the precise sample characteristics were reported in the supplementary information. Since we could not activate the hyperlink to this information, we contacted the authors. They provided us with the supplement, and with additional information on the number of singleton and multiplex families, involved in their study.
- In the study by Guerini et al. (2006) [11], the reported data on the transmission of short and long 5-HTTLPR variants by heterozygous parents to affected offspring were not fully clear to us, so we contacted the authors for clarification. They provided us with the applicable information, and with additional information on the number of singleton and multiplex families, involved in their study.
- Ultra-long (XL) 5-HTTLPR variants, included in two studies [12,13], were considered long – as in the original studies.
- The values that we found for χ^2 and p with regard to the study by Koishi et al. (2006) [14] turned out to deviate substantially from the values of these variables as originally published. Upon request, the authors informed us that this was due to the fact that they applied Yates's correction [15] for continuity to their data. Indeed, we could therewith reproduce the values they reported for χ^2 and p. However, for reasons of consistency we did not apply this correction to our own χ^2- and p-values in the context of this meta-analysis.

For the other studies, discrepancies were absent or considered to be attributable to round-off.

Table S1. Applicable Items of the PRISMA (Preferred Reporting Items for Systematic Reviews and Meta-Analyses) Checklist [16] and Their Locations in the Manuscript

Item	Location
**TITLE & ABSTRACT**	
Title, identifying the report as a systematic review and meta-analysis	heading
Structured summary, including background, objectives, data sources, synthesis methods, results, conclusions and implications of key findings	paragraph 1
**BACKGROUND**	
Description of the rationale for the review in the context of what is already known	paragraph 1–6
Explicit statement of questions being addressed	paragraph 7
**METHODS**	
Specification of study and report characteristics used as criteria for eligibility, with rationale	**Selection Criteria**
Description of information sources in the search and date last searched	**Search Strategy**
Presentation of full electronic search strategy for at least one database, including any limits used	**Data Extraction**
Description of the process for selecting studies, included in systematic review and meta-analysis	**Selection Criteria**
Description of the method of data extraction from reports and processes for obtaining and confirming data from investigators	**Search Strategy**
List of all variables for which data were sought	**Data extraction**
Definition of the principal summary measures	**Synthesis of Results**
Description of the methods of combining results of studies, including measures of consistency, for each meta-analysis	**Statistical Analysis,** paragraph 1–2
Specification of assessment of risk of bias that may affect the cumulative evidence	**Statistical Analysis,** paragraph 2
Description of a method for additional analyses, indicating that it was pre-specified	**Statistical Analysis,** paragraph 3
**RESULTS**	
Description of numbers of studies screened, assessed for eligibility, and included in the review, with reasons for exclusions at each stage, with a flow diagram	**Search Results;** page S1; **Figure S1**
Description of characteristics for which data were extracted for each study, including citations	**Table 1**
Presentation, for each study and for all outcomes considered:(a) simple summary data (b) effect estimates and confidence intervals	**Table 1; Figure 2**
Presentation of results of each meta-analysis done, including confidence intervals and measures of consistency	**Primary Data and Synthesis of results; Table 1; Figure 2**
Presentation of results of the assessments of risk of bias across studies	**Across Study Bias; Figure S2, S3**
Description of results of additional analysis	**Sensitivity Analysis**
**DISCUSSION & CONCLUSIONS**	
Summary of the main findings, including the strength of evidence for each main outcome, and consideration of their relevance to key groups	paragraph 1–3; **Relevance**
Discussion of limitations at outcome level and at review-level	**Strengths and Limitations**
General interpretation of the results in the context of other evidence, and implications for future research	**Suggestions for Future Research; CONCLUSIONS**

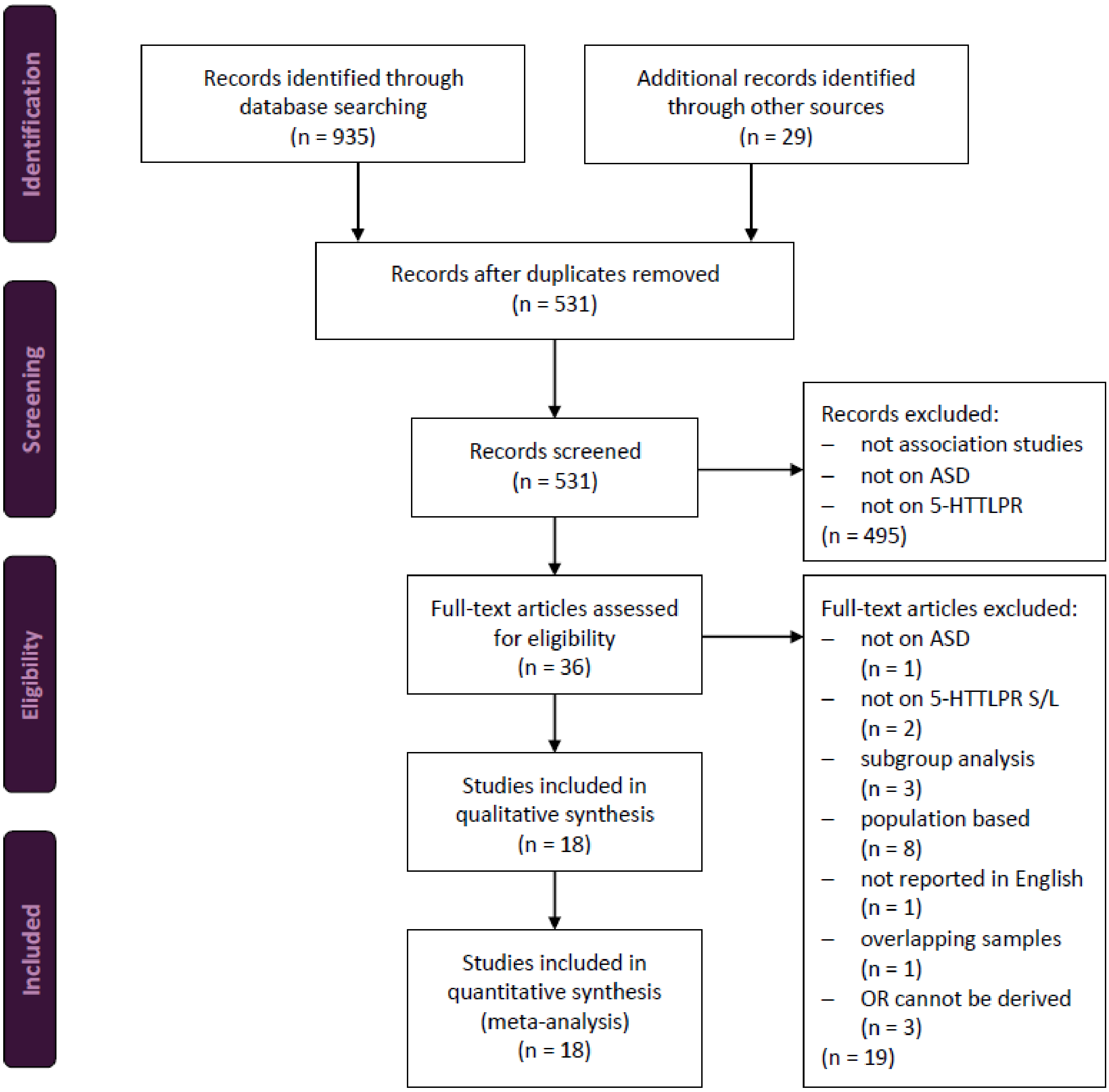

Figure S1. PRISMA flow diagram [16]. Results of literature search of transmission disequilibrium test (TDT) studies on the association between the short/long (S/L) 5-HTTLPR polymorphism and autism spectrum disorder (ASD). 18 studies, reported in 17 articles, were included in our meta-analysis. OR, odds ratio.

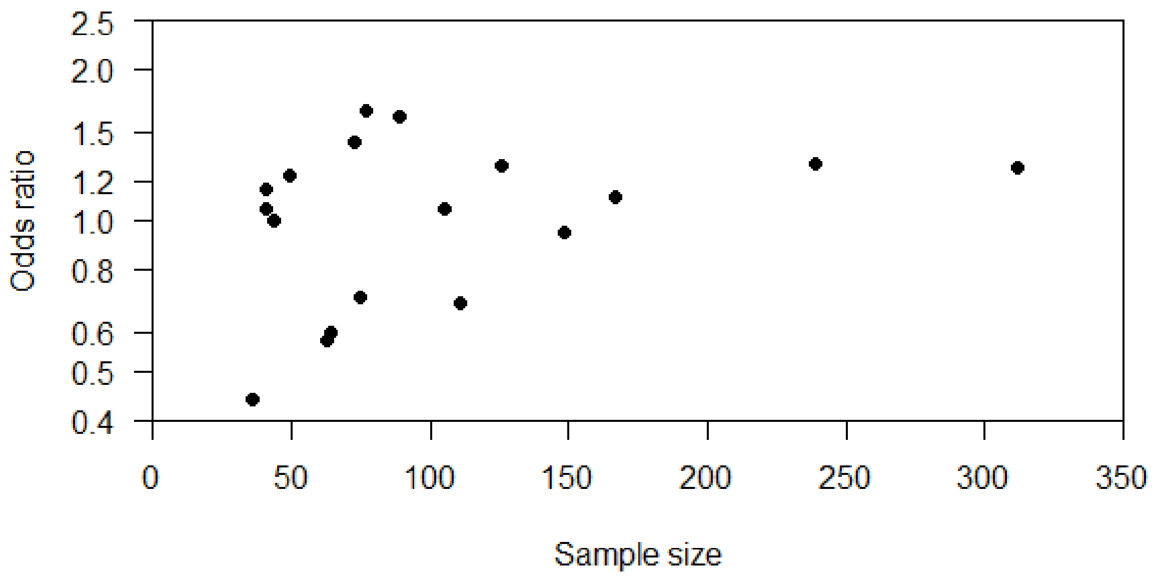

Figure S2. Funnel plot [17,18] of the estimated odds ratios as a function of sample size

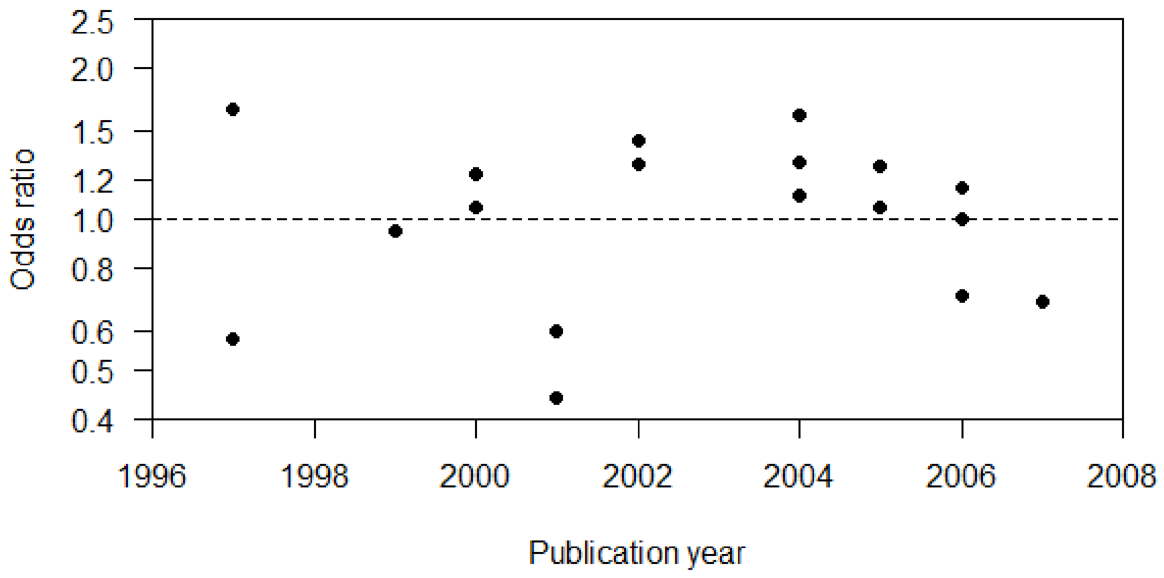

Figure S3. Result of a meta-regression of odds ratio on year of publication

Supplementary References

1. Persico, A.M.; Militerni, R.; Bravaccio, C.; Schneider, C.; Melmed, R.; Conciatori, M.; Damiani, V.; Baldi, A.; Keller, F. Lack of association between serotonin transporter gene promoter variants and autistic disorder in two ethnically distinct samples. *Am. J. Med. Genet.* **2000**, *96*, 123–127. [CrossRef]
2. Valencia, A.V.; Páez, A.L.; Sampedro, M.E.; Ávila, C.; Cardona, J.C.; Mesa, C.; Galvis, L.; Carrizosa, J.; Camargo, M.; Ruiz, A.; et al. Evidence for association and epistasis between the genetic markers SLC6A4 and HTR2A in autism etiology. *Biomedica* **2012**, *32*, 585–601. [CrossRef]
3. Coutinho, A.M.; Sousa, I.; Martins, M.; Correia, C.; Correia, C.; Morgadinho, T.; Bento, C.; Marques, C.; Ataíde, A.; Miguel, T.S.; et al. Evidence for epistasis between SLC6A4 and ITGB3 in autism etiology and in the determination of platelet serotonin levels. *Hum. Genet.* **2007**, *121*, 243–256. [CrossRef]
4. Coutinho, A.M.; Oliveira, G.; Morgadinho, T.; Fesel, C.; Macedo, T.R.; Bento, C.; Marques, C.; Ataíde, A.; Miguel, T.; Borges, L. Variants of the serotonin transporter gene (SLC6A4) significantly contribute to hyperserotonemia in autism. *Mol. Psychiatry* **2004**, ***9***, 264–271. [CrossRef]
5. Ramoz, N.; Reichert, J.G.; Corwin, T.E.; Smith, C.J.; Silverman, J.M.; Hollander, E.; Buxbaum, J.D. Lack of evidence for association of the serotonin transporter gene SLC6A4 with autism. *Biol. Psychiatry* **2006**, *60*, 186–191. [CrossRef]
6. Longo, D.; Schüler-Faccini L.; Brandalize AP.; dos Santos Riesgo R.; Bau CH. Influence of the 5-HTTLPR polymorphism and environmental risk factors in a Brazilian sample of patients with autism spectrum disorders. *Brain Res.* **2009**, *1267*, 9–17. [CrossRef]
7. Jaiswal, P.; Guhathakurta, S.; Singh, A.S. *SLC6A4* markers modulate platelet 5-HT level and specific behaviors of autism: a study from an Indian population. *Prog. Neuropsychopharmacol. Biol. Psychiatry* **2015**, *56*, 196–206. [CrossRef]
8. McCauley, J.L.; Olson, L.M.; Dowd, M.; Amin, T.; Steele, A.; Blakely, R.D.; Folstein, S.E..; Haines J.L.; Sutcliffe, J.S. Linkage and association analysis at the serotonin transporter (SLC6A4) locus in a rigid-compulsive subset of autism. *Am. J. Med. Genet. B Neuropsychiatr. Genet.* **2004,** *127B*, 104–112. [CrossRef]
9. Huang, C.H.; Santangelo, S.L. Autism and serotonin transporter gene polymorphisms: a systematic review and meta-analysis. *Am. J. Med. Genet. B Neuropsychiatr. Genet.* **2008**, *147B*, 903–913. [CrossRef]
10. Mulder, E.J.; Anderson, G.M.; Kema, I.P.; Brugman, A.M.; Ketelaars, C.E.; de Bildt, A.; van Lang, N.D.; den Boer, J.A.; Minderaa, R.B. Serotonin transporter intron 2 polymorphism associated with rigid-compulsive behaviors in Dutch individuals with pervasive developmental disorder. *Am. J. Med. Genet. B Neuropsychiatr. Genet.* **2005**, *133B*, 93–96. [CrossRef]
11. Guerini, F.R.; Manca, S.; Sotgiu, S.; Tremolada, S.; Zanzottera, M.; Agliardi, C.; Zanetta, L.; Saresella, M.; Mancuso, R.; De Silvestri, A.; et al. A family based linkage analysis of HLA and 5-HTTLPR gene polymorphisms in Sardinian children with autism spectrum disorder. *Hum. Immunol.* **2006**, *67*, 108–117. [CrossRef]
12. Cho, I.H.; Yoo, H.J.; Park, M.; Lee, Y.S.; Kim, S.A. Family-based association study of 5-HTTLPR and the 5-HT2A receptor gene polymorphisms with autism spectrum disorder in Korean trios. *Brain Res.* **2007**, *1139*, 34–41. [CrossRef]
13. Guhathakurta, S.; Ghosh, S.; Sinha, S.; Chatterjee, A.; Ahmed, S.; Chowdhury, S.R. Gangopadhyay, P.K, Ghosh, S.; Singh, M.; Usha, R. Serotonin transporter promoter variants: Analysis in Indian autistic and control population. *Brain Res.* **2006**, *1092*, 28–35. [CrossRef]
14. Koishi, S.; Yamamoto, K.; Matsumoto, H.; Koishi, S.; Enseki, Y.; Oya, A.; Asakura, A.; Aoki, Y.; Atsumi, M.; Iga T.; et al. Serotonin transporter gene promoter polymorphism and autism: A family-based genetic association study in Japanese population. *Brain Dev.* **2006**, *28*, 257–260. [CrossRef]
15. Yates, F. Contingency tables involving small numbers and the χ2 test. *J. Roy. Stat. Soc. Suppl.* **1934**, *1*, 217–235. [CrossRef]
16. Moher, D.; Liberati, A.; Tetzlaff, J.; Altman, D.G. Preferred Reporting Items for Systematic Reviews and Meta-Analyses: The PRISMA Statement. *PLoS Med.* **2009**, *6*, e1000097. [CrossRef]
17. Begg, C.B.; Mazumdar, M. Operating characteristics of a rank correlation test for publication bias. *Biometrics* **1994**, *50*, 1088–1101. [CrossRef]

18. Light, R.J.; Singer, J.D.; Willett, J.B. The Visual Presentation and Interpretation of Meta-Analyses. In *The Handbook of Research Synthesis*.; Cooper, H., Hedges, L.V., Eds.; Russell Sage Foundation: New York, United States, **1994**; pp 439–453.
